# Emergent zero-field anomalous Hall effect in a reconstructed rutile antiferromagnetic metal

**DOI:** 10.1038/s41467-023-43962-0

**Published:** 2023-12-12

**Authors:** Meng Wang, Katsuhiro Tanaka, Shiro Sakai, Ziqian Wang, Ke Deng, Yingjie Lyu, Cong Li, Di Tian, Shengchun Shen, Naoki Ogawa, Naoya Kanazawa, Pu Yu, Ryotaro Arita, Fumitaka Kagawa

**Affiliations:** 1 RIKEN Center for Emergent Matter Science (CEMS), Wako, 351-0198 Japan; 2 Research Center for Advanced Science and Technology, University of Tokyo, Tokyo, 153-8904 Japan; 3 Shenzhen Institute for Quantum Science and Engineering, Southern University of Science and Technology (SUSTech), Shenzhen, 518055 China; 4 International Quantum Academy, Shenzhen, 518048 China; 5 State Key Laboratory of Low Dimensional Quantum Physics and Department of Physics, Tsinghua University, Beijing, 100084 China; 6 Department of Physics, University of Science and Technology of China, Hefei, 230026 China; 7 Department of Applied Physics and Quantum-Phase Electronics Center (QPEC), University of Tokyo, Tokyo, 113-8656 Japan; 8 Institute of Industrial Science, The University of Tokyo, Tokyo, 153-8505 Japan; 9 Department of Physics, Tokyo Institute of Technology, Tokyo, 152-8551 Japan

**Keywords:** Magnetic properties and materials, Electronic properties and materials

## Abstract

The anomalous Hall effect (AHE) that emerges in antiferromagnetic metals shows intriguing physics and offers numerous potential applications. Magnets with a rutile crystal structure have recently received attention as a possible platform for a collinear-antiferromagnetism-induced AHE. RuO_2_ is a prototypical candidate material, however the AHE is prohibited at zero field by symmetry because of the high-symmetry [001] direction of the Néel vector at the ground state. Here, we show AHE at zero field in Cr-doped rutile, Ru_0.8_Cr_0.2_O_2_. The magnetization, transport and density functional theory calculations indicate that appropriate doping of Cr at Ru sites reconstructs the collinear antiferromagnetism in RuO_2_, resulting in a rotation of the Néel vector from [001] to [110] while maintaining a collinear antiferromagnetic state. The AHE with vanishing net moment in the Ru_0.8_Cr_0.2_O_2_ exhibits an orientation dependence consistent with the [110]-oriented Hall vector. These results demonstrate that material engineering by doping is a useful approach to manipulate AHE in antiferromagnetic metals.

## Abstract (web summary)

The anomalous Hall effect is typically associated with ferromagnets and referred to as anomalous due to its persistence even after the applied magnetic field is removed, due to the net magnetization of the ferromagnet. Recently there has been much interest in antiferromagnets that can host an anomalous Hall effect, despite a vanishing magnetization, and here, Wang et al observe an anomalous Hall effect in collinearly antiferromagnetic chromium doped RuO_2_.

## Introduction

The Anomalous Hall effect (AHE) long considered as a unique feature of ferromagnetic metals, and its magnitude was empirically taken as proportional to the macroscopic magnetization *M*^1,2^. It followed that in antiferromagnetic materials, which host zero macroscopic magnetization or only small canting moments, the AHE should be negligibly small. However, recent theoretical works indicate that in some antiferromagnetic materials, the AHE can be expected if the magnetic space group (MSG) (or, equivalently, the magnetic point group that the MSG belongs to) allows for a nonzero Berry curvature and/or asymmetric scattering, even if the corresponding macroscopic magnetization is zero^3–5^. Such an AHE was first demonstrated for various noncollinear antiferromagnets with magnetic multipoles^6–12^, such as kagome Mn_3_Sn and pyrochlore *R*_2_Ir_2_O_7_.

**Fig. 1:**
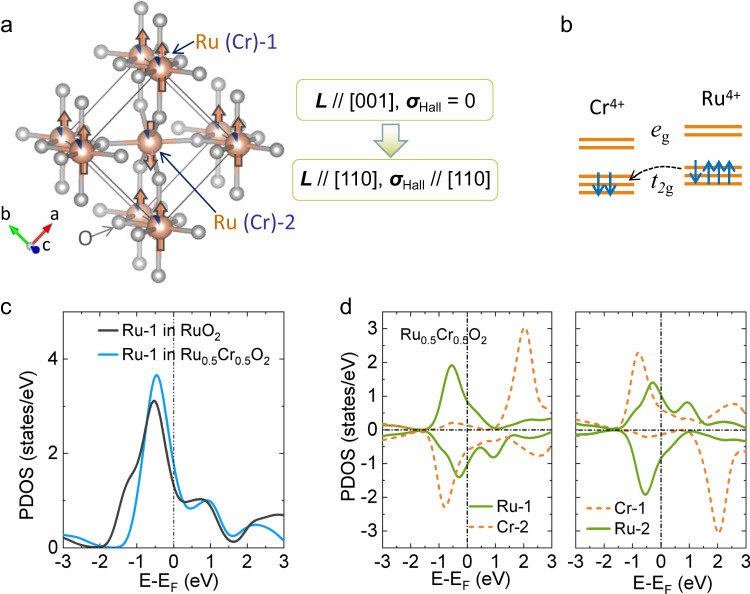
Antiferromagnetic symmetry controlled anomalous Hall effect (AHE) and DFT calculations for Cr-doped RuO_2_. **a** Crystal structure of the Cr-doped rutile phase RuO_2_. O-ions are located between two Ru (Cr) sites asymmetrically. The Ru-1 (Cr-1) and Ru-2 (Cr-2) denote the Ru (Cr) ions at the center and the corner sites of the unit cell, respectively. The orange arrows denote the local magnetic moment with antiferromagnetic coupling along [110]. Hall vector (***σ***_Hall_) is allowed and parallel to the Néel vector (***L***) along [110] in such a configuration, which vanishes as the Néel vector is along [001], indicating a manipulating of ***L*** is necessary to generate AHE. **b** Schematic illustration of charge transfer in Cr-doped RuO_2_. The orbital level difference between the nearest neighbor sites can lead to partial charge transfer from Ru^4+^ to Cr^4+^ to form a reconstructed Fermi level and maintain an antiparallel spin coupling. **c** Calculated projected density of states (PDOS) of the RuO_2_ and Ru_0.5_Cr_0.5_O_2_ in the paramagnetic phase. The Ru-2 sites for both components possess identical PDOS with Ru-1. **d** Calculated PDOS of the Ru_0.5_Cr_0.5_O_2_ in the magnetic ground state. The doped Cr ions have two selective sites as labeled by Cr-1 and Cr-2 in (**a**). Ru and Cr both show an asymmetric PDOS (a spontaneous polarization), while exhibiting an antiparallel coupling.

From the symmetry point of view, an antiferromagnetism-induced AHE can also be expected in a collinear antiferromagnet. Recently, such a concept has been proposed in a series of materials^13–15^ and experimentally observed in the collinear antiferromagnetic semiconductor MnTe^16^. Among these materials, RuO_2_, which has a rutile structure and exhibits a collinear antiferromagnetic order, has received significant attention as a model system of the antiferromagnetism-induced AHE^17–20^. As shown in Fig. 1a, the crystal structure of RuO_2_ consists of two Ru sublattices with antiparallel magnetic moments. The two magnetic sublattices have different chemical environments due to the asymmetric O–Ru–O bond configuration. The simplest argument to determine the presence or absence of the AHE under collinear antiferromagnetism would be to consider how the Hall vector ***σ***_Hall_ = (σ_yz_, σ_zx_, σ_xy_) is transformed by the symmetry operations^14,18^; here, note that σ_yz_, σ_zx_, and σ_xy_ represent only anti-symmetric part of the conductivity tensor. When the Néel vector (***L***) of RuO_2_ is along the [110] direction, the MSG is C*mm’m’*, in which ***σ***_Hall_ along [110] is invariant under all symmetry operations and thus allows for a zero-field AHE^14^. In contrast, if ***L*** || [001], the MSG is P4*’*_*2*_/*mnm’*, which does not allow for a finite ***σ***_Hall_ because no vector can be invariant under two orthogonal rotation symmetry operations (see Supplementary Note 1 for details)^14^. A previous neutron diffraction indicates that the Néel vector in RuO_2_ is along [001]^17^, and hence ***σ***_Hall_ and the zero-field AHE are prohibited by symmetry (Supplementary Fig. 1).

To unveil the AHE associated with the collinear antiferromagnetism in RuO_2_, a recent study focused on tilting the Néel vector from [001] toward [110] by utilizing a high magnetic field of ~50 T^19,20^. This phenomenon can be viewed as a magnetic-field-induced AHE associated with a Néel vector, forming a sharp contrast to AHEs in ferromagnets^1,2^, in which the AHE can be observed even under zero field. Achieving a zero-field AHE in such a rutile-type collinear antiferromagnet remains a major challenge for experiments.

Previous density functional theory (DFT) calculations have revealed that the easy axis of the Néel vector in RuO_2_ sensitively depends on the electron filling^19,20^, which inspired us to pursue the zero-field AHE in the derivatives of RuO_2_ by means of appropriate modulations on its Fermi level. To change the direction of the Néel vector from [001] and therefore render the zero-field AHE allowed by symmetry, we dope Cr into RuO_2_. Since the 4*d* orbital level of Ru^4+^ is slightly higher than the 3*d* orbital level of Cr^4+^, a charge transfer from Ru^4+^ to Cr^4+^ ions is expected (Fig. 1b)^21,22^ while favouring antiparallel spin coupling between the nearest-neighboring Ru and Cr sites. Considering that collinear spin orders are realized in both RuO_2_ (antiferromagnetic) and CrO_2_ (ferromagnetic) in rutile phases^17,23,24^, the collinear antiferromagnetic state is reasonably expected in stoichiometric proximity to RuO_2_.

It should be noted that the collinear antiferromagnetism in RuO_2_ has been questioned quite recently^25^. Nevertheless, considering that many previous experiments support the collinear antiferromagnetism^17,19,24,26,27^, we designed the experiment by postulating that the magnetism of RuO_2_ at the ground state is a collinear antiferromagnetic state with the Néel vector along [001] and interpret the experimental results with the assumption that a small amount of Cr-doping does not change the collinear antiferromagnetism but can modulate the direction of the Néel vector. Within this approach, our magnetometry suggests that the direction of the Néel vector in the Ru_0.8_Cr_0.2_O_2_ film shifts to [110]. Concomitantly, we find that the Ru_0.8_Cr_0.2_O_2_ film exhibits an appreciable zero-field AHE with hysteretic behavior while the net magnetization is vanishingly small. These observations are well explained by considering that the collinear antiferromagnetism with the Néel vector along [110] is realized and that the magnetic field switches the two collinear antiferromagnetic states that are related by time-reversal operation.

## Results

### DFT calculations on the impact of Cr-doping

To gain insight into the impact of Cr-doping on the Fermi level, we first performed DFT calculations for the paramagnetic states of Ru_1-x_Cr_x_O_2_ for x = 0 and 0.5. As shown in Fig. 1c, by doping Cr, the shift of the projected density of states (or, equivalently, the shift of the Fermi level) is observed, as expected. The magnetic calculation for x = 0.5 (Fig. 1d) further demonstrates that the ground state has appreciable local magnetic moments with antiparallel couplings among the nearest-neighboring Cr and Ru ions. Note that the DFT + *U* calculations on RuO_2_ show that the energy difference with the Néel vector orienting to [001], [100], and [110] is tiny (~5 meV) and that the easy-axis direction sensitively depends on the Fermi level (Supplementary Fig. 2)^19^. Our DFT results therefore support our working hypothesis that Cr doping is a promising approach to change the Néel vector direction while maintaining the collinear antiferromagnetic order.

The DFT+DMFT results indicate that Cr doping is also accompanied by the enhancement of the local magnetic moment. For the case of non-doped RuO_2_, the Ru ions exhibit a negligibly small spin polarization when *U* is small (<1 eV) (Supplementary Fig. 3). In contrast, when Cr is doped, considerable local moments are observed (0.15 μ_B_ for x = 0.25 and 0.4 μ_B_ for x = 0.5; see Supplementary Fig. 3) in the DFT calculations, even at *U* = 0.

Thus, based on our DFT calculations, we can expect that the easy axis of the Néel vector changes from the original [001] direction, in which the zero-field AHE is prohibited. These expectations are verified by the experiments described below.

### Films fabrication and valence evaluation

We synthesized the Ru_1-x_Cr_x_O_2_ films by pulsed laser deposition (PLD) on TiO_2_ (110) substrates with x = 0.1, 0.2, and 0.3 (see “Methods”). The high crystalline quality of the films was confirmed by X-ray 2*θ-ω* scans (see supplementary Fig. 4a) and the surface topography with atomic terraces (Supplementary Fig. 4c). Besides, the resistivities of the materials increase as the doping level increases, while all compounds show a metallic behavior, as shown in Supplementary Fig. 5a. The robust metallicity implies the strong overlap of Cr and Ru orbitals.

**Fig. 2:**
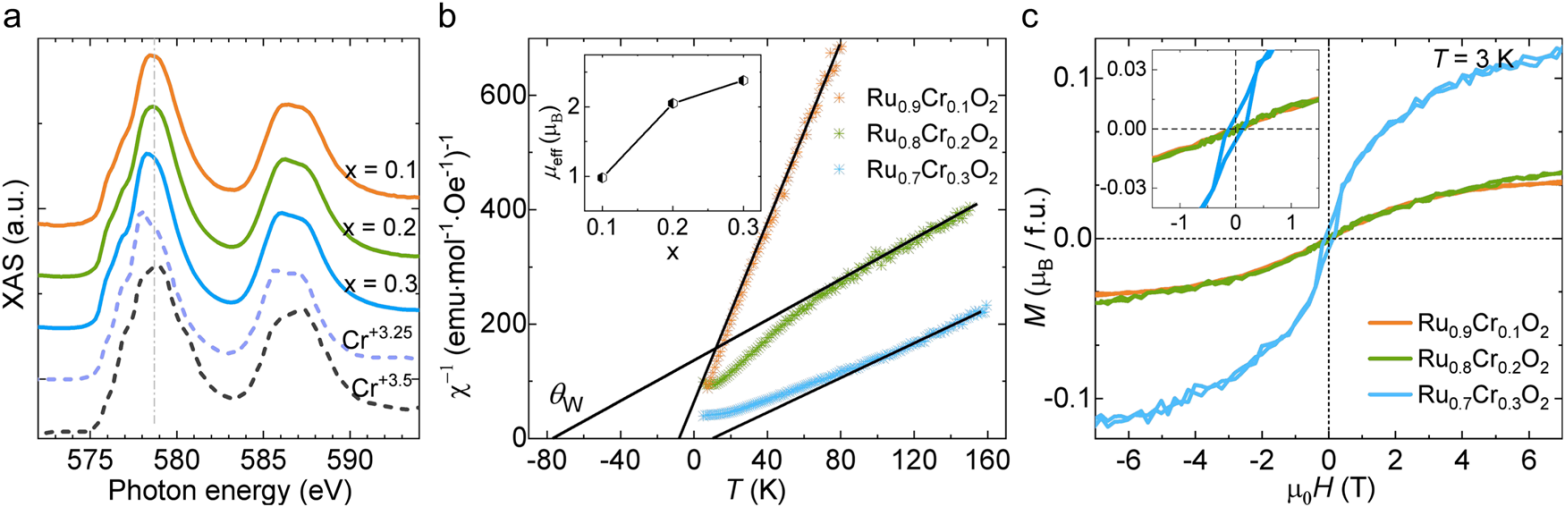
XAS and magnetic states evolution in Ru_1-x_Cr_x_O_2_ films grown on TiO_2_ (110). **a** XAS around the *L*-edge of Cr measured in the Ru_1-x_Cr_x_O_2_ films compared to that in La_1-x_Sr_x_CrO_3_^28^. **b**, **c** Temperature-dependent magnetic susceptibility (**b**) and magnetic field-dependent magnetization (**c**) curves measured with a magnetic field along the out-of-plane (OOP) axis. All films are grown on TiO_2_ (110). Inset of (**b**), the effective on-site moments (*μ*_eff_) depending on the doping level x. Inset of (**c**), an expanded view of the low-field region. Linear fittings of the χ^−1^–*T* curves at high temperatures indicate an antiferromagnetic behavior with negative Weiss temperatures *θ*_W_ = −10 K and −75 K in x = 0.1 and 0.2, respectively. The x = 0.3 film shows a small positive *θ*_W_, with a finite remanent magnetization at zero field, implying a ferrimagnetic ground state. The magnetic field is 1 T for the χ^−1^–*T* measurement.

To probe the valence state of the doped Cr in the rutile lattice, we carried out soft X-ray absorption spectroscopy (XAS) measurements (see “Methods”) on the three films. Figure 2a shows the XAS results near the *L*-edge of Cr, with a comparison to that from La_1-x_Sr_x_CrO_3_ materials^28^. The Cr in all of the Ru_1-x_Cr_x_O_2_ films exhibits a fractional valence state between +3.25 and +3.5. As the doping level increases from 0.1 to 0.3, the peak shows a gradual shift to lower energy, indicating a gradual decrease in valence. Such a result is consistent with our scenario that the Cr doping is accompanied by the charge transfer and the corresponding Fermi-level shift.

### Antiferromagnetic metal phases in the Ru_1-x_Cr_x_O_2_ films

To check whether the magnetic ground state is still antiferromagnetic upon the Cr doping, we performed magnetic susceptibility (χ) and magnetization (*M*) measurements with magnetic field (*H*) and temperature (*T*) dependences (see “Methods” and Supplementary Fig. 6 for details). The results are summarized in Figs. 2b, c, and we first focus on the results of x = 0.1 and 0.2. The high-temperature regions of the χ^−1^‒*T* profiles are fitted with the Curie‒Weiss law, χ = *C*/(*T*–*θ*_W_), and we obtain *θ*_W_ ≈ ‒10 K and ‒75 K for x = 0.1 and 0.2, respectively. These results indicate that an antiferromagnetic interaction is dominant in x = 0.1 and 0.2^29–32^. Moreover, the effective on-site moments (*μ*_eff_) obtained from the fittings are distinctly enhanced with increasing Cr-doping levels (Fig. 2b, inset and Supplementary Note 2)^29,30^, which is also consistent with our DFT calculations.

The *M*‒*H* curves at the lowest temperature, 3 K, demonstrate that the spontaneous net magnetization at zero field is too small to be distinguished in the antiferromagnetic Ru_0.9_Cr_0.1_O_2_ and Ru_0.8_Cr_0.2_O_2_ (Fig. 2c). Moreover, the field-induced moment at 7 T is only 0.03 μ_B_ (x = 0.1) and 0.04 μ_B_ (x = 0.2) per formula unit (μ_B_/f.u.), which are almost two orders of magnitude smaller than that in ferromagnetic SrRuO_3_ and CrO_2_^33–35^, excluding the possibility of a ferromagnetic ground state for x = 0.1 and 0.2.

In the Ru_0.7_Cr_0.3_O_2_ film, contrastingly, the analysis based on the Curie‒Weiss law results in a small positive *θ*_W_ with *μ*_eff_ of ~2.5 μ_B_ per site (Fig. 2b, and Supplementary Note 2). Furthermore, the *M*‒*H* curve exhibits a finite remanent magnetization, and the magnetization at 7 T is distinctly larger compared with the case of x = 0.1 and 0.2. These observations indicate the evolution of a ferrimagnetic phase in x = 0.3, consistent with the tendency from RuO_2_ to CrO_2_^17,23,24,26^. Therefore, the AHE accompanying the ferrimagnetic phase in x = 0.3 is beyond the scope of this study.

### Néel-vector direction in the Ru_0.8_Cr_0.2_O_2_ film

**Fig. 3:**
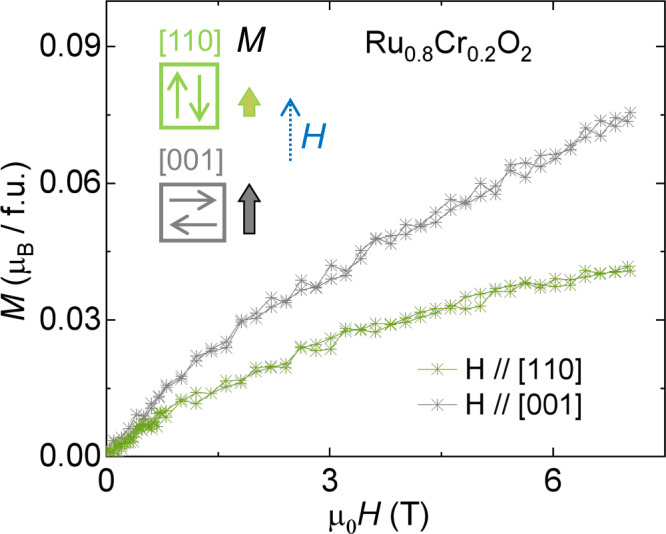
Magnetic anisotropy and Néel vector orientation in Ru_0.8_Cr_0.2_O_2_ film grown on TiO_2_ (110). *M*–*H* curves were measured at 3 K with magnetic field along out-of-plane (*H* || [110]) and in-plane (*H* || [001]), respectively. Inset, the illustration of spin orientation and corresponding moments (wide arrows) driven by the external magnetic field (*H*) applied to [110] and [001].

We then focus on the antiferromagnetic Ru_0.8_Cr_0.2_O_2_ (110) sample and aim to reveal the direction of the Néel vector. The DFT calculations in RuO_2_ suggest a finite net magnetic moment when the Néel vector along [100] is assumed (Supplementary Fig. 2a), which should be preserved in the doped phase. Our *M*–*H* measurements in Ru_0.8_Cr_0.2_O_2_ show a vanishing net moment, thereby ruling out the possibility that the Néel vector is along [100]. Then, the remaining candidates of the Néel-vector direction are the [001] and [110] orientations. To test these two possibilities, we refer to the fact that the field-induced moment in a collinear antiferromagnet is generally minimized when the field is parallel to the Néel vector, as illustrated in Fig. 3 inset^19,30^. The anisotropy of the field-induced moment was measured on the Ru_0.8_Cr_0.2_O_2_ (110) film for the fields of the out-of-plane [110] and in-plane [001] directions. The anisotropic response demonstrates that the [110] axis exhibits a smaller field-induced moment (Fig. 3), suggesting that the Néel vector is likely along [110], rather than [001], in Ru_0.8_Cr_0.2_O_2_, although other orientations cannot be ruled out completely. Note that if the Néel vector along [110] is realized, the zero-field AHE is allowed by symmetry^14,19^. This is verified by the transport measurements below.

### AHE in the Ru_0.8_Cr_0.2_O_2_ (110) film

The longitudinal resistivity and Hall conductivity in Ru_0.8_Cr_0.2_O_2_ (110) film were measured with currents along two in-plane directions, [001] and $[1\bar{1}0]$, as shown in Supplementary Fig. 5 (see “Methods”). Both directions show a metallic state, and the anomalous Hall conductivity (AHC) measured with the current along $[1\bar{1}0]$ exhibits a larger signal. Therefore, we below present the results of the AHE with the current along $[1\bar{1}0]$.

**Fig. 4:**
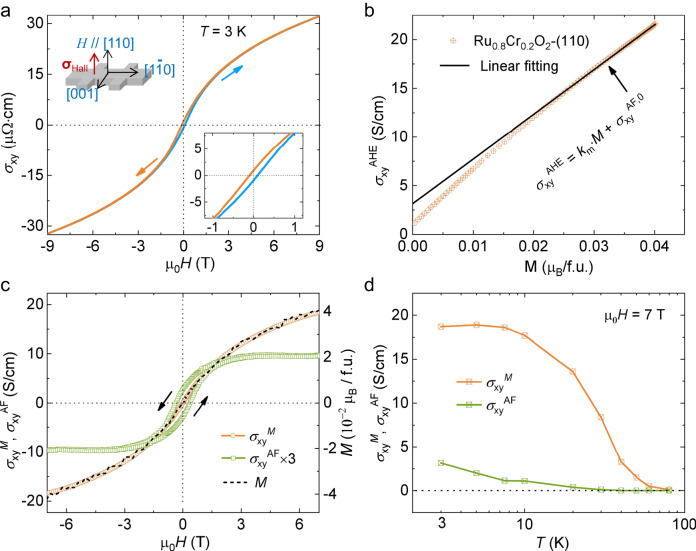
Transport properties of the Ru_0.8_Cr_0.2_O_2_ film grown on TiO_2_ (110). **a** Hall conductivity with magnetic field dependence at 3 K. Insets show the Hall configuration (left) and an expanded view of the low-field region (right). ***σ***_Hall_, Hall vector. **b** Anomalous Hall conductivity at 3 K with a dependence on the magnetic moment (*M*). The σ_xy_^AHE^ was obtained by subtracting a field-linear-dependent ordinary Hall contribution from σ_xy_. *M* was measured by an MPMS at 3 K. **c** Anomalous Hall conductivity derived from the canting moment (i.e., σ_xy_^M^) and the antiferromagnetic domain (i.e., σ_xy_^AF^) in Ru_0.8_Cr_0.2_O_2_ (110) with a dependence on magnetic field sweeping at 3 K. The magnetic moment is shown by a dashed line. **d** Temperature-dependent σ_xy_^M^ and σ_xy_^AF^. The data at 7 T are used.

Figure 4a shows the Hall conductivity (σ_xy_) with a magnetic field sweeping at 3 K. Distinctly, a hysteretic feature is observed, in stark contrast to the absence of a hysteretic behavior in the *M*–*H* curve (Fig. 2c). This behavior demonstrates that the finite Hall vector is involved in the Ru_0.8_Cr_0.2_O_2_ (110) film, even though the net magnetization is vanishingly small within the experimental accuracy. Thus, in the magnetic field range in which σ_xy_ shows hysteretic behavior, one should take into account the coexistence of the two magnetic domains with opposite Hall vectors (i.e., the AHCs with opposite signs).

In general, the origin of σ_xy_ consists of the external magnetic field (or ordinary Hall conductivity, σ_xy_^OHE^, proportional to *H* with a coefficient *k*_*o*_) and the magnetism (or anomalous Hall conductivity, σ_xy_^AHE^). The σ_xy_^AHE^ is often dictated by the contribution proportional to the net magnetization, but in the present system, the antiferromagnetic order coupled with the special lattice symmetry can also contribute^3,6,14^. Thus, the observed σ_xy_ can be described as the sum of the three components:

1 $${\upsigma }_{{{{{{\rm{xy}}}}}}}(H)={{\upsigma }_{{{{{{\rm{xy}}}}}}}}^{{{{{{\rm{OHE}}}}}}}(H)+{{\upsigma }_{{{{{{\rm{xy}}}}}}}}^{M}(H)+{{\upsigma }_{{{{{{\rm{xy}}}}}}}}^{{{{{{\rm{AF}}}}}}}(H)={k}_{o}\cdot H+{k}_{m}\cdot M(H)+{{\upsigma }_{{{{{{\rm{xy}}}}}}}}^{{{{{{\rm{AF}}}}}}}(H),$$

where σ_xy_^*M*^ is the anomalous Hall conductivity proportional to the field-induced net magnetic moment *M* with a coefficient *k*_*m*_, and σ_xy_^AF^ is the anomalous Hall conductivity arising from the antiferromagnetic ordering^6^. Note that in the present field range, the magnetic field-dependent σ_xy_^AF^(*H*) is caused by the change in the relative volume of the two types of antiferromagnetic domains with opposite signs of AHC.

At sufficiently high magnetic fields, the hysteretic behavior disappears, and therefore, a single antiferromagnetic domain is expected. Thus, σ_xy_^AF^ is considered to be a constant, σ_xy_^AF,0^, at a sufficiently high magnetic field^14^. Utilizing the data of Hall conductivity and magnetization at 4‒7 T, where the hysteretic behavior is absent, we can thus obtain the coefficients, *k*_*o*_ and *k*_m_, and σ_xy_^AF,0^. For clarity, by subtracting σ_xy_^OHE^ = *k*_*o*_·*H*, we display the experimental σ_xy_^AHE^ together with the fitting curve *k*_*m*_·*M* + σ_xy_^AF,0^ as a function of the net magnetization in Fig. 4b. The value of σ_xy_^AF,0^ is ≈3.2 S/cm, which is indicated by the intercept of the fitting curve at *M* = 0. In the low-field region, the experimental σ_xy_^AHE^(*H*) deviates from the linear fitting. In the present framework, this deviation is attributable to the coexistence of two antiferromagnetic domains with opposite signs of AHC.

The evolutions of σ_xy_^AF^ and σ_xy_^*M*^ with magnetic field sweeping at 3 K are shown in Fig. 4c, where σ_xy_^*M*^ is set to *k*_*m*_·*M*, and σ_xy_^AF^ is obtained by subtracting σ_xy_^*M*^ from σ_xy_^AHE^. Interestingly, the σ_xy_^AF^ shows a hysteretic profile and a clear remnant value even at the vanishing net moment (Fig. 2c). Such features indicate an AHC contributed by the antiferromagnetic ordering, not due to the canting moment. The emergent σ_xy_^AF^ decreases as the temperature increases and disappears at 40–50 K (Fig. 4d and Supplementary Fig. 7), indicating the antiferromagnetic order transition point (*T*_N_).

To gain further insight into the microscopic mechanisms of the σ_xy_^AF^ and σ_xy_^*M*^, we compared the AHC‒σ_xx_ scaling curves^2,36–39^ among Ru_0.8_Cr_0.2_O_2_ (110) films with different σ_xx_, which was tuned by tailoring the thickness. As shown in Supplementary Fig. 8, all films are located at the crossover from dirty to intermediate regimes with 10^3^ <σ_xx_ < 10^4 ^S/cm, thereby ruling out the skew scattering contribution, which is generally considered in high conductive metals (σ_xx_ > 10^6 ^S/cm). Besides, a further analysis based on the σ_xy_^*M*^ (*T*)‒σ_xx_(*T*)^*2*^ profile gives an intrinsic Berry curvature term of 14 S/cm (Supplementary Note 3) and the extrinsic side-jump contribution of ~10 S/cm. These results indicate that the Berry curvature and extrinsic scattering microscopic mechanisms both contributes to σ_xy_^*M*^ (*T*) in our films. We note that the σ_xy_^*M*^ value is similar to the AHC in ferromagnetic SrRuO_3_ films grown by PLD, although the canting moment (0.04 μ_B_/f.u.) of our Ru_0.8_Cr_0.2_O_2_ (110) film is ~40 times smaller than the ferromagnetic moment in SrRuO_3_ films^40,41^. We also note that the value of σ_xy_^AF^ in Ru_0.8_Cr_0.2_O_2_ is one order of magnitude larger than the recently reported collinear antiferromagnetic semiconductor MnTe^16^.

### Orientation-anisotropic anomalous Hall response

**Fig. 5:**
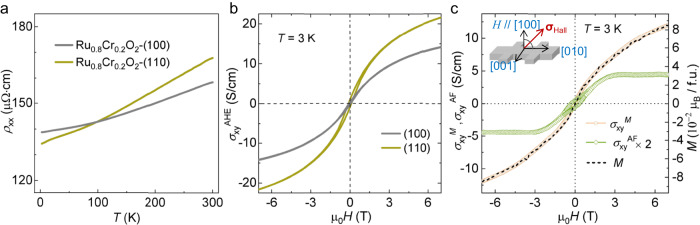
Comparison of the transport behaviors for films grown along (100) and (110). **a** Temperature-dependent longitudinal resistivities of the two films. **b** Magnetic field-dependent σ_xy_^AHE^ at 3 K for the two films. **c** σ_xy_^M^ and σ_xy_^AF^ with magnetic-field dependence at 3 K for the film grown along the (100) orientation. The magnetic moment is shown by a dashed line. During the transport measurement on the Ru_0.8_Cr_0.2_O_2_-(100) film, the current was applied along the [010] direction with a Hall voltage along the [001] direction for comparison. Inset, the illustration of the Hall bar and the Hall vector (***σ***_Hall_).

Finally, we show that the transport properties in our Ru_0.8_Cr_0.2_O_2_ film also indicate the Hall vector along [110]. To address this issue experimentally, we referred to the fact that the transverse anomalous Hall current ( ***J***_H_) is given by ***J***_H_ = ***E*** × ***σ***_Hall_^14,18^, where ***E*** represents the applied external electric field, and carried out transport measurements on another film grown on TiO_2_ (100). Herein, the current was applied along the [010] direction to keep the Hall voltage also along the [001] direction for comparison. The temperature dependence of AHE is similar to that observed for the [110]-oriented films (Supplementary Fig. 7), indicating that the transition temperature is not affected by the orientation of the substrate. As shown in Fig. 5a, b, the longitudinal conductivities at low temperatures of the two films are very close to each other, while the σ_xy_^AHE^ that emerges from the (100) film is distinctly smaller than the value for the film grown on TiO_2_ (110). Upon further analyzing the magnetization and the anomalous Hall contributions of σ_xy_^*M*^ and σ_xy_^AF^, as shown in Fig. 5c, we find that both of the anomalous Hall components are suppressed compared with those in Fig. 4c. Furthermore, we find that the saturated σ_xy_^AF^ in the [100]-oriented film is approximately ~2.2 S/cm, which is 0.7 (≃sin45°) times that in the [110]-oriented sample, ~3.2 S/cm. These transport results further support that the Hall vector is directed along the [110] direction in this compound, as illustrated in Fig. 5c, inset.

## Discussion

To support the assumed collinearity of the antiferromagnetism, it may be useful to refer to the fact that the noncollinear antiferromagnetic materials with frustrated spin interactions are prone to show a large value of |*θ*_W_/*T*_N_| (>10)^42^. In our Ru_0.8_Cr_0.2_O_2_ film, the value of |*θ*_W_/*T*_N_| is found to be small, 1.5‒1.8, which at least does not contradict the assumed collinearity. While our experimental observations in the Ru_0.8_Cr_0.2_O_2_ films can be consistently understood by assuming that the collinear antiferromagnetism with the Néel vector along [110] is realized, more rigorous evidence of the magnetic structure, for instance using neutron diffraction, remains a challenge in the future studies.

In summary, by inducing Fermi-level shift, we have succeeded in changing the easy axis of the Néel vector from that of RuO_2_ and thus observing the zero-field AHE in the reconstructed collinear antiferromagnetic rutile metal, Ru_0.8_Cr_0.2_O_2_. While the antiferromagnetic metallic phase is rare in correlated oxides^31,32,43^, our study indicates a possibility to broaden the candidate materials and produce variations of the antiferromagnetic Néel vector by doping. We envision that this material-design strategy may also work and be helpful to explore the AHE in other rutile oxides, such as ReO_2_^44^.

## Methods

### DFT calculations and Wannierization

We computed the Bloch wavefunctions for RuO_2_ on the basis of density functional theory (DFT) using the Quantum ESPRESSO package^45,46^. We first assumed a nonmagnetic structure without spin-orbit coupling and used the projector augmented wave pseudopotential^47^ and the generalized gradient approximation of the Perdew–Burke–Ernzerhof exchange correlation functional^48^. We used lattice constants of a = 4.492 Å and c = 3.107 Å. The energy cutoff for the wave function and the charge density, *e*_wfc_ and *e*_rho_, respectively, were set to *e*_wfc_ = 60 Ry and *e*_rho_ = 400 Ry. We used ***k***-point meshes of 12 × 12 × 16 and 16 × 16 × 16 in the self-consistent field (scf) and non-scf calculations, respectively. After the DFT calculations, Wannierization was performed by using the wannier90 package^49,50^, in which the Bloch orbitals were projected onto the *t*_2g_ orbitals of Ru ions with 16 × 16 × 16 ***k***-point grids.

To calculate the electronic states of Ru_1-x_Cr_x_O_2_, with x = 0, 0.25, and 0.5, we replaced the Ru-sites denoted as Ru-1 or Ru-2 in Supplementary Fig. 3a with Cr. In this calculation, we set *e*_rho_ = 500 Ry, and the spin-orbit coupling was not included. For the x = 0 and 0.5 systems, we took 24 × 24 × 32 ***k***-mesh for the scf calculation. When we calculated the ground states of Ru_0.75_Cr_0.25_O_2_, we used the supercell with the b- or c-axis doubled. We took the ***k***-mesh of 24 × 12 × 32 (24 × 24 × 16) when the b- (c-)axis was doubled for the scf calculation. We found that the supercell with the b-axis doubled was more energetically stable, which we have used for discussion. To obtain the projected density of states (PDOS) of the x = 0 and 0.5 systems, we performed the non-scf calculations with 24 × 24 × 32 ***k***-mesh after the scf calculation and then calculated the PDOS. We also calculated the PDOS of RuO_2_ with the DFT + *U* method with *U* = 3 eV and nonmagnetic Ru_1-x_Cr_x_O_2_ with x = 0 and 0.5, where we set *e*_rho_ = 500 Ry and took 24 × 24 × 32 ***k***-points for the scf and non-scf calculations.

For examining the orientation of the Néel vector, we performed the DFT + *U* calculation for RuO_2_ with the spin-orbit coupling for the three cases where the Néel vector was initially along [001], [100], and [110]. We took *U* = 3 eV. We used 24 × 24 × 32 ***k***-points and set *e*_rho_ = 500 Ry. The convergence threshold for the calculation of the Néel vector orientation was set as 10^−6 ^Ry.

### DMFT calculations

The Wannier functions obtained above define a tight-binding model for the three Ru *t*_2g_ orbitals of RuO_2_. Using this as the one-body part of the Hamiltonian, we constructed a multiorbital Hubbard model with intra(inter)orbital Coulomb interaction *U*(*U’)* and Hund’s coupling and pair hopping *J*. We solved the model within the dynamical mean field theory (DMFT)^51^ at zero temperature. As a solver for the DMFT impurity problem, we used the exact diagonalization method^52^, where the dynamical mean field was represented by nine bath sites. To obtain the antiferromagnetic solution, we assumed opposite spin polarizations at neighboring Ru sites in the unit cell. For the interaction parameters, we assumed *U* = *U’* + 2*J* and *J* = *U*/5 for the sake of simplicity.

### Thin-film growth, X-ray diffraction, and XAS

The Ru_1-x_Cr_x_O_2_ films were grown on the rutile TiO_2_ substrate by the PLD method with stoichiometric targets. During sample growth, the substrate temperature was kept at 290 °C to suppress interfacial diffusion, and the oxygen partial pressure was kept at 20 mTorr. The laser fluence was 1.2 J/cm^2^ (KrF, λ = 248 nm), and the deposition frequency was 3 Hz. After deposition, the samples were cooled to room temperature at a rate of 10 °C/min under an oxygen pressure of 10 Torr. The film thickness was determined directly with an X-ray reflectivity measurement. X-ray diffraction measurements were performed using a high-resolution diffractometer (Rigaku) with monochromatic Cu K_α1_ (λ = 1.5406 Å) X-rays. The stoichiometry in the thin film was checked by energy dispersive X-ray (EDX), and the ratio of Ru/Cr was confirmed to be very close to the target. The XAS curves of Cr L-edge were measured with a total electron mode, at 20 K, in beamline BL07U of Shanghai Synchrotron Radiation Facility.

### Transport and magnetization measurements

All of the electrical transport was carried out on Hall bar devices with a size of 300 μm × 60 μm, which were fabricated by photolithography. The milling process was carried out with Ar/O_2_ (10:1) mixed ions and at a low speed to avoid oxygen vacancy formation on the TiO_2_ surface. The transport measurements were carried out with a PPMS system (Quantum Design) with an in-plane DC current. The magnetoresistivity (MR) and its anisotropy were very small, as shown in Supplementary Fig. 9. The Hall conductivity σ_xy_ was calculated as σ_xy_ = −*ρ*_yx_/(*ρ*_xx_^2^). The magnetization was measured using an MPMS system (Quantum Design) and obtained by subtracting the contribution from the TiO_2_ substrate. The Hall vector (***σ***_Hall_) is defined as that in ref. ^14^.

### Supplementary information

Supplementary Information

Peer Review File

## Data Availability

All data used to generate the figures in the manuscript and supplementary information is available on Zenodo at: https://zenodo.org/record/8412950.
