# Supplementary material for: Emergent zero-field anomalous Hall effect in a reconstructed rutile antiferromagnetic metal
Source: Nat Commun. 2023 Dec 12;14:8240. doi: 10.1038/s41467-023-43962-0 (PMC10716394; doi:10.1038/s41467-023-43962-0)
Supplement: Supplementary file 1 — Supplementary Information [file 41467_2023_43962_MOESM1_ESM.pdf]

Supplementary Information for

Emergent zero-field anomalous Hall effect in a reconstructed rutile antiferromagnetic metal

Meng Wang[1,*,†], Katsuhiro Tanaka[2,†], Shiro Sakai[1], Ziqian Wang[1], Ke Deng[3,4], Yingjie Lyu[5], Cong Li[5], Di Tian[5], Shengchun Shen[6], Naoki Ogawa[1,7], Naoya Kanazawa[8], Pu Yu[5], Ryotaro Arita[1,2], Fumitaka Kagawa[1,9,*]

[1]*RIKEN Center for Emergent Matter Science (CEMS), Wako, 351-0198, Japan.*

[2]*Research Center for Advanced Science and Technology, University of Tokyo, Tokyo 153-8904, Japan.*

[3]*Shenzhen Institute for Quantum Science and Engineering, Southern University of Science and Technology (SUSTech), Shenzhen 518055, China.*

[4]*International Quantum Academy, Shenzhen 518048, China.*

[5]*State Key Laboratory of Low Dimensional Quantum Physics and Department of Physics, Tsinghua University, Beijing, 100084, China.*

[6]*Department of Physics, University of Science and Technology of China, Hefei, 230026, China.*

[7]*Department of Applied Physics and Quantum-Phase Electronics Center (QPEC), University of Tokyo, Tokyo 113-8656, Japan.*

[8]*Institute of Industrial Science, The University of Tokyo, Tokyo 153-8505, Japan.*

[9]*Department of Physics, Tokyo Institute of Technology, Tokyo 152-8551, Japan.*

[*]Corresponding authors. Email: meng.wang@riken.jp; fumitaka.kagawa@riken.jp

[†]These authors contributed equally: Meng Wang, Katsuhiro Tanaka.

Supplementary Note 1, Symmetry analysis of AHE for rutile RuO_2.

Supplementary Note 2, Curie-Weiss law fitting and calculation of effective moment.

Supplementary Note 3, Distinguish the side-jump and intrinsic anomalous Hall conductivity.

Supplementary Table 1, Spin order and moments of $Ru_{0.75}Cr_{0.25}O_2$ calculated with DFT with the b-axis doubled cell.

Supplementary Table 2, Spin order and moments calculated with DFT for $Ru_{0.5}Cr_{0.5}O_2$.

Supplementary Fig. 1, AHE in RuO_2 films grown on TiO_2.

Supplementary Fig. 2, DFT+U calculations on the anisotropy of pure RuO_2.

Supplementary Fig. 3, Calculated results for $Ru_{1-x}Cr_xO_2$.

Supplementary Fig. 4, Structure characteristics and surface morphology of the $Ru_{1-x}Cr_xO_2$ films.

Supplementary Fig. 5, **Transport properties in $Ru_{1-x}Cr_xO_2$ (110) and the x = 0.2 film with current along different directions.**

Supplementary Fig. 6, **Magnetization measurements in a $Ru_{0.8}Cr_{0.2}O_2$ (110) film.**

Supplementary Fig. 7, **Magnetic-field dependent Hall conductivity at various temperatures**.

Supplementary Fig. 8, **Scaling of $\sigma_{xy}^M \sim \sigma_{xx}$ in the $Ru_{0.8}Cr_{0.2}O_2$ (110) films**.

Supplementary Fig. 9, **Magnetoresistivity (MR) measured for the $Ru_{0.8}Cr_{0.2}O_2$ (110) film**.

Supplementary Note 1, Symmetry analysis of AHE for rutile RuO_2:

The Hall vector $\boldsymbol{\sigma}_{\mathrm{Hall}} = (\sigma_{yz}, \sigma_{zx}, \sigma_{xy})$, is defined as in reference 14. The RuO_2 with the Néel vector along the [001] direction has a magnetic space group (MSG) of $P4_2'/mnm'$. The $P4_2'/mnm'$ has $\boldsymbol{t}_{1/2}\boldsymbol{C}_{2x}$ and $\boldsymbol{t}_{1/2}\boldsymbol{C}_{2y}$ symmetry operations, where $\boldsymbol{C}_{2x}$ ($\boldsymbol{C}_{2y}$) is an operation of rotating 180° along the x (y) axis and $\boldsymbol{t}_{1/2}$ denotes a translation (a/2, b/2, c/2), which correspond to $\boldsymbol{C}_{2x}$ and $\boldsymbol{C}_{2y}$ in the magnetic point group (MPG). Furthermore, $\boldsymbol{C}_{2x}(\sigma_{yz}, \sigma_{zx}, \sigma_{xy}) = (\sigma_{yz}, -\sigma_{zx}, -\sigma_{xy})$ and $\boldsymbol{C}_{2y}(\sigma_{yz}, \sigma_{zx}, \sigma_{xy}) = (-\sigma_{yz}, \sigma_{zx}, -\sigma_{xy})$, demonstrate $\boldsymbol{\sigma}_{\mathrm{Hall}} = \mathbf{0}$; i.e., σ_{yz}, σ_{zx},and σ_{xy} are all prohibited. In contrast, when the Néel vector is along [100] and [110], the MSG is *Pnn'm'* and *Cmm'm'*, respectively. The MPG symmetry operations in *Pnn'm'* include $\boldsymbol{TC}_{2x}$, $\boldsymbol{C}_{2y}$ and $\boldsymbol{TC}_{2z}$, where $\boldsymbol{T}$ represents the time-reversal operation; i.e., $\boldsymbol{TC}_{2x}(\sigma_{yz}, \sigma_{zx}, \sigma_{xy}) = (-\sigma_{yz}, \sigma_{zx}, \sigma_{xy})$ and $\boldsymbol{TC}_{2z}(\sigma_{yz}, \sigma_{zx}, \sigma_{xy}) = (\sigma_{yz}, \sigma_{zx}, -\sigma_{xy})$. Thus, only σ_{zx} can be allowed. Given the fact that *Pnn'm'* and *Cmm'm'* consider different principal axes, one can conclude that the Hall vector is finite and directed along [010] (when the Néel vector || [100]) and [110] (when the Néel vector || [110]).

Supplementary Note 2, Curie-Weiss law fitting and calculation of effective moment:

According to the Curie-Weiss law, the magnetic order can be checked utilizing the relationship of magnetic susceptibility (χ) and absolute temperature (T) at the temperature regions above the magnetic ordering transitions, by:[29,30]

$$\chi = C / (T - \theta_W) \qquad (1)$$

where C is the Curie constant and θ_W is often referred to as the Weiss temperature (or Weiss constant). The fittings of Equation (1) with a positive, negative, and zero of θ_W indicate the ferromagnetic, antiferromagnetic, and paramagnetic phases, respectively. Furthermore, the constant C can be given by:

$$C = N_A \cdot \mu_0 \cdot \mu_{eff}^2 / (3k_B) \qquad (2)$$

where the $\mu_{eff}^2 = g_J (J+1) J \cdot \mu_B^2$, k_B is Boltzmann's constant, N_A the Avogadro constant, g_J the Landé g-factor, μ_B the Bohr magneton, J the angular momentum quantum number.

The Equation (2) calculated with the Gaussian units can give a: [29]

$$\mu_{eff} = (8C)^{1/2}\,\mu_B \ \ [cgs] \qquad (3)$$

The $\chi^{-1} \sim T$ curves were fitted to obtain the C constants, and then the μ_{eff} were calculated.

Supplementary Note 3, Distinguish the side-jump and intrinsic anomalous Hall conductivity:

We try to distinguish the side-jump and intrinsic contributions of the σ_{xy}^M in the high conductivity region, which is generally considered through the following equation: [36-39]

$$\sigma_{xy}^{AHE} = -(\alpha\, \sigma_{xx0}^{-1} + \beta\, \sigma_{xx0}^{-2})\, M\, \sigma_{xx}(T)^2 + b\, M \qquad (4)$$

Where, the M is the magnetization, [$\alpha\, \sigma_{xx0}^{-1}\, M\, \sigma_{xx}(T)^2$], [$\beta\, \sigma_{xx0}^{-2}\, M\, \sigma_{xx}(T)^2$], and ($b\, M$) correspond to the anomalous anomalous Hall conductivity due to skew scattering, side-jump, and intrinsic mechanisms, respectively. The skew scattering in such a regime is negligible ($\alpha \approx 0$). We then carried out the fitting of $\sigma_{xy}^M \sim \sigma_{xx}(T)^2$ for the 10 nm, 20 nm, and 40 nm films respectively, as shown in Supplementary Fig. 9. Considering the M does not decrease too much with temperature increasing, the data of 3 K to 20 K were used. Note that the conductivity does not change too much within such a temperature region, and we can only obtain an estimation with the fitting by Equation (4), and an intrinsic anomalous Hall conductivity (σ_{xy}^{int}) of ~14 S/cm is obtained.

Supplementary Table 1, Spin order and moments of $Ru_{0.75}Cr_{0.25}O_2$ calculated with DFT with the b-axis doubled cell.

Cr at Ru-1 site			Cr at Ru-2 site	
Cr-1	1.8456 (μ_B)		Ru-1	0.1912 (μ_B)
Ru-2	-0.1911		Cr-2	-1.8456
Ru-1	0.0642		Ru-1	0.1912
Ru-2	-0.1911		Ru-2	-0.0642

Supplementary Table 2, Spin order and moments calculated with DFT for $Ru_{0.5}Cr_{0.5}O_2$.

Cr at Ru-1 site			Cr at Ru-2 site	
Cr-1	1.8434 (μ_B)		Ru-1	0.4031 (μ_B)
Ru-2	-0.4023		Cr-2	-1.8436

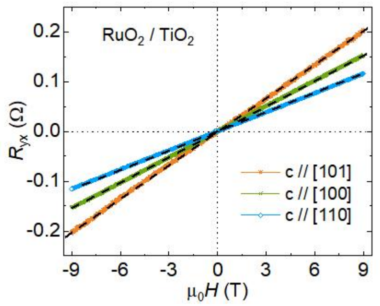

Supplementary Fig. 1, AHE in RuO_2 films grown on TiO_2. Magnetic field dependent Hall resistances (R_{yx}) of the RuO_2 films grown on TiO_2 with c axis along [100], [110], and [101] at 3 K.

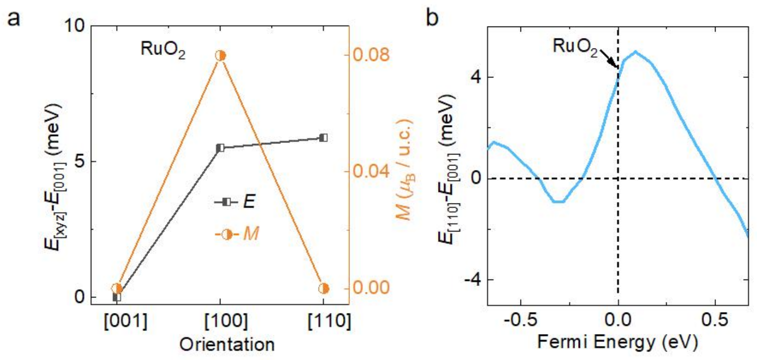

Supplementary Fig. 2, DFT+U calculations on the anisotropy of pure RuO_2. a, Calculated energy (E) and net moment (M) in pure RuO_2 with U = 3 eV depending on the initial orientation of the Néel vector. The state with Néel vector along [001] exhibits the lowest energy and no spontaneous moment. The states with Néel vector along [100] or [110] show about 5 meV higher energy, while the [100] case generates a spontaneous moment of 0.08 μ_B / unit cell. **b**, Calculated energy difference, $E_{[110]}$-$E_{[001]}$, in pure RuO_2 depending on the Fermi level. Data are obtained from reference 19.

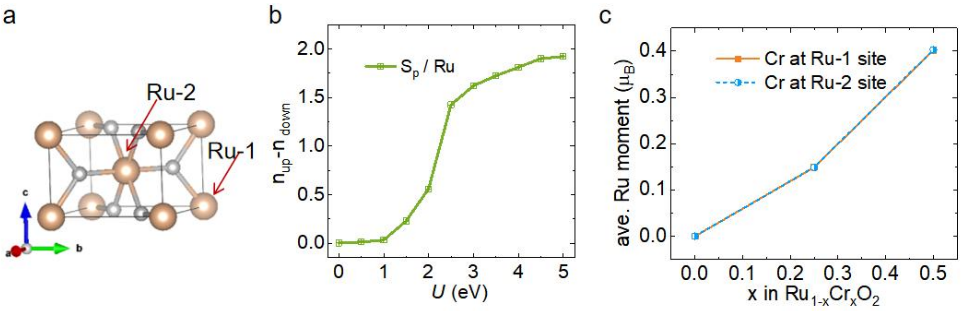

Supplementary Fig. 3, **Calculated results for $Ru_{1-x}Cr_xO_2$. a**, Crystal structure of one unit cell of RuO_2, where the two Ru sites are labeled as Ru-1 and Ru-2. **b**, DFT+DMFT result of the spin polarization (S_p=n_{up}-n_{down}) of RuO_2, plotted against the onsite Coulomb interaction U. For a small U (< 1 eV), S_p is almost negligible while it increases distinctly around U=2-3 eV. **c**, Average Ru moment with respect to the Cr doping level x in $Ru_{1-x}Cr_xO_2$, calculated with DFT.

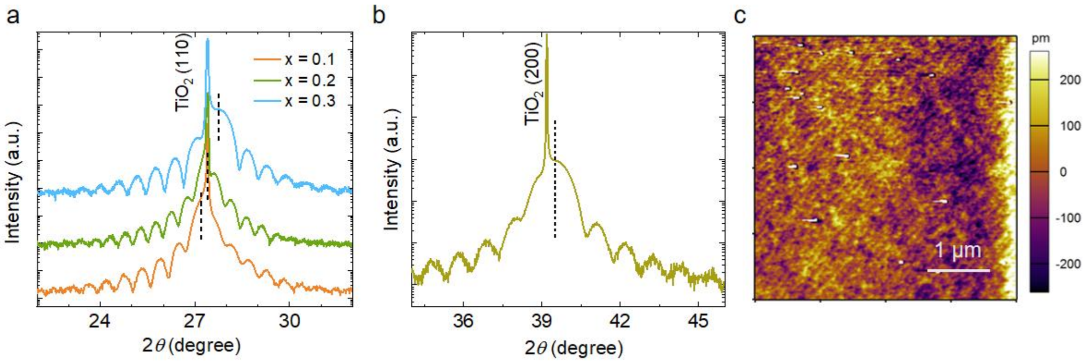

Supplementary Fig. 4, **Structure characteristics and surface morphology of the $Ru_{1-x}Cr_xO_2$ films**. **a**, XRD 2θ-ω curves of $Ru_{1-x}Cr_xO_2$ with x = 0.1, 0.2 and 0.3, measured around the TiO_2 (110). **b**, 2θ-ω curve of the $Ru_{0.8}Cr_{0.2}O_2$ film grown on TiO_2 (200). The diffraction peaks of the films are indicated by the d lines. **c**, surface morphology of the $Ru_{0.8}Cr_{0.2}O_2$ (110) film measured by atomic force microscopy (AFM).

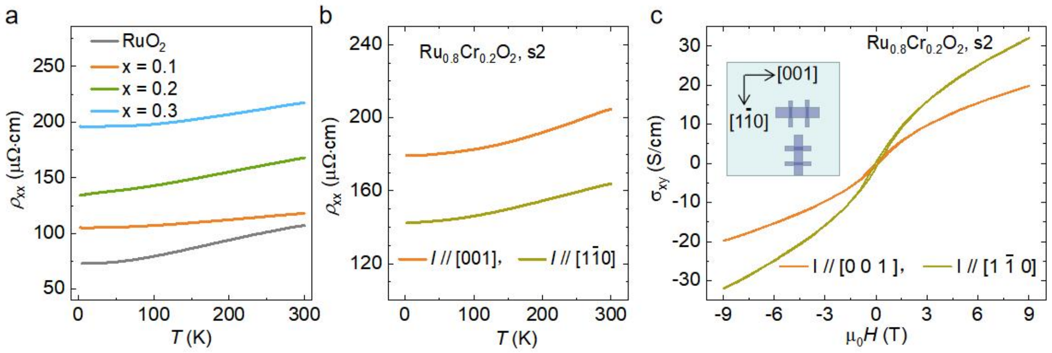

Supplementary Fig. 5, **Transport properties in $Ru_{1-x}Cr_xO_2$ (110) and the x = 0.2 film with current along different directions**. **a**, Temperature-dependent resistivities (*RT*) curves in $Ru_{1-x}Cr_xO_2$ films grown on TiO_2 (110). **b**, *RT* curves in $Ru_{0.8}Cr_{0.2}O_2$ measured with current along two orientations. **c**, σ_{xy} with magnetic field sweeping at 3 K in two current directions. The inset shows the configuration of the Hall measurements. S2, Sample No. 2 of the $Ru_{0.8}Cr_{0.2}O_2$ film.

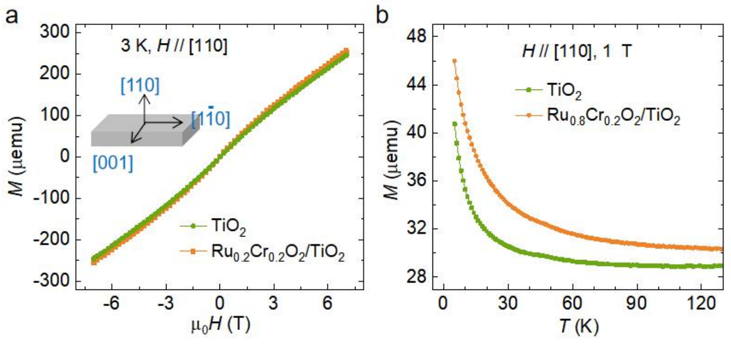

Supplementary Fig. 6, **Magnetization measurements in a $Ru_{0.8}Cr_{0.2}O_2$ (110) film.** **a**, **b**, Raw data for magnetization in the $Ru_{0.8}Cr_{0.2}O_2/TiO_2$ sample and a pure TiO_2 substrate with magnetic field (a) and temperature (b) dependence. The inset in (A) illustrates the crystal orientation of the film. The film thickness is 55 nm.

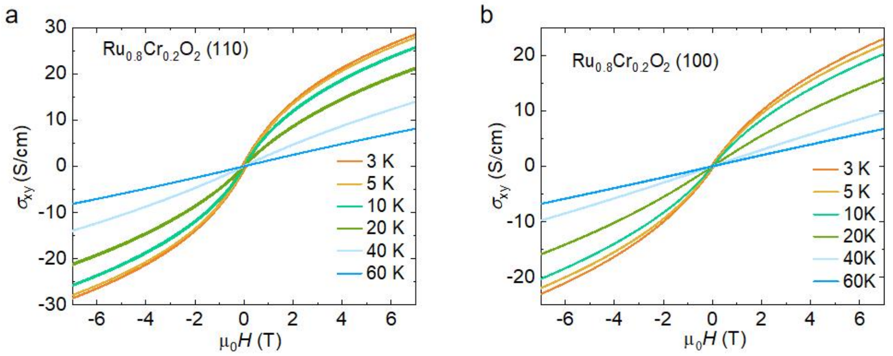

Supplementary Fig. 7, Magnetic-field dependent Hall conductivity at various temperatures. σ_{xy} with magnetic field dependence measured at a series of temperatures for $Ru_{0.8}Cr_{0.2}O_2$ films grown along the (110) and (100) orientations.

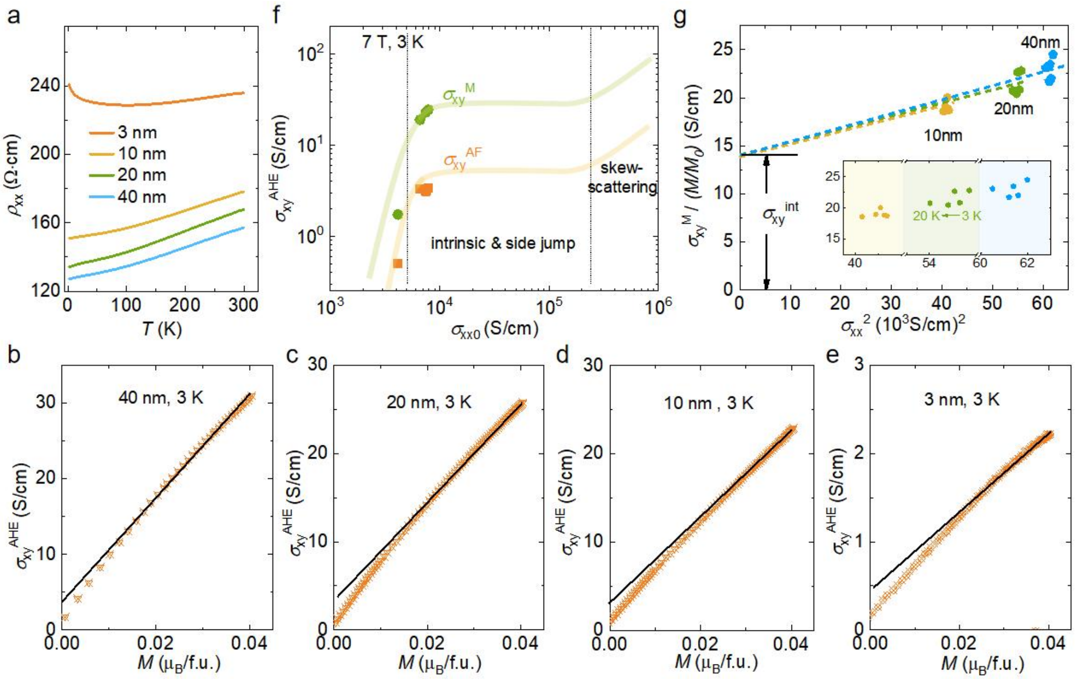

Supplementary Fig. 8, Scaling of σ_{xy}^{M} ~ σ_{xx} in the $Ru_{0.8}Cr_{0.2}O_2$ (110) films. Evolution of anomalous Hall conductivity with the longitudinal conductivity from an itinerant metal to a localized state in $Ru_{0.8}Cr_{0.2}O_2$. **a**, *RT* curves of the $Ru_{0.8}Cr_{0.2}O_2$ films with thickness changing. **b-e**, Fittings of the σ_{xy}^{AHE} - M curves at high field regions to obtain the σ_{xy}^{M} and σ_{xy}^{AF} components for the films with a thickness of 40 nm (b), 20 nm (c), 10 nm (d), and 3 nm (e). **f**, Evolution of σ_{xy}^{M} and σ_{xy}^{AF} with the changing of σ_{xx0}. σ_{xx0} are set by the conductivity at 3 K. Dash lines denoting the boundaries of two regions, are given by reference 37. **g**, Linear fitting of the

normalized σ_{xy}^{M} - $\sigma_{xx}(T)^2$ with the data of 3 K - 20 K used for the 10 nm, 20 nm, and 40 nm films, respectively. The intercept denotes the intrinsic term of anomalous Hall conductivity. Inset, a zoom-in of the data.

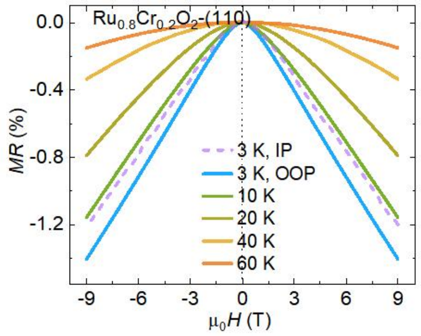

Supplementary Fig. 9, Magnetoresistivity (MR) measured for the $Ru_{0.8}Cr_{0.2}O_2$ (110) film. Out-of-plane (OOP) MR curves were measured at a series of temperatures and compared to the in-plane (IP) MR at 3 K. The IP field was along [001]. The current was along $[1\bar{1}0]$ for all measurements.
